# Capturing and Selecting Senescence Variation in Wheat

**DOI:** 10.3389/fpls.2021.638738

**Published:** 2021-04-16

**Authors:** Elizabeth A. Chapman, Simon Orford, Jacob Lage, Simon Griffiths

**Affiliations:** ^1^Department of Crop Genetics, John Innes Centre, Norwich Research Park, Norwich, United Kingdom; ^2^KWS UK Ltd., Thriplow, United Kingdom

**Keywords:** senescence, phenotyping, selection, staygreen, grain development, peduncle, leaves, quantification

## Abstract

Senescence is a highly quantitative trait, but in wheat the genetics underpinning senescence regulation remain relatively unknown. To select senescence variation and ultimately identify novel genetic regulators, accurate characterization of senescence phenotypes is essential. When investigating senescence, phenotyping efforts often focus on, or are limited to, the visual assessment of flag leaves. However, senescence is a whole-plant process, involving remobilization and translocation of resources into the developing grain. Furthermore, the temporal progression of senescence poses challenges regarding trait quantification and description, whereupon the different models and approaches applied result in varying definitions of apparently similar metrics. To gain a holistic understanding of senescence, we phenotyped flag leaf and peduncle senescence progression, alongside grain maturation. Reviewing the literature, we identified techniques commonly applied in quantification of senescence variation and developed simple methods to calculate descriptive and discriminatory metrics. To capture senescence dynamism, we developed the idea of calculating thermal time to different flag leaf senescence scores, for which between-year Spearman’s rank correlations of *r* ≥ 0.59, *P* < 4.7 × 10^–5^ (TT70), identify as an accurate phenotyping method. Following our experience of senescence trait genetic mapping, we recognized the need for singular metrics capable of discriminating senescence variation, identifying thermal time to flag leaf senescence score of 70 (TT70) and mean peduncle senescence (MeanPed) scores as most informative. Moreover, grain maturity assessments confirmed a previous association between our staygreen traits and grain fill extension, illustrating trait functionality. Here we review different senescence phenotyping approaches and share our experiences of phenotyping two independent recombinant inbred line (RIL) populations segregating for staygreen traits. Together, we direct readers toward senescence phenotyping methods we found most effective, encouraging their use when investigating and discriminating senescence variation of differing genetic bases, and aid trait selection and weighting in breeding and research programs alike.

## Introduction

**FIGURE 1:**
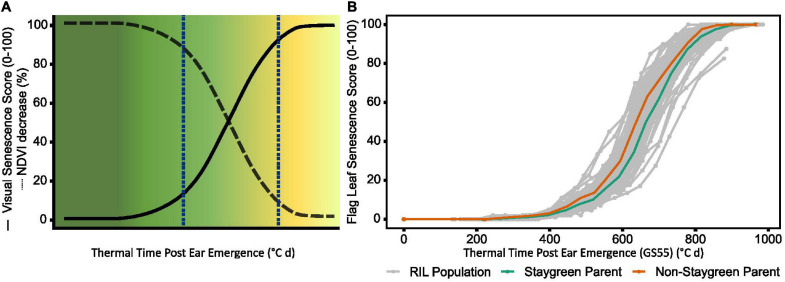
During the rapid senescence phase flag leaves transition from green to yellow **(A)**, with inter-line variation sometimes difficult to distinguish **(B)**. Senescence is scored on a 0–100 scale based in progression of leaf yellowing (visual) or greenness reduction (NDVI-based). Scores are plotted against thermal time post-anthesis (°C day) to standardize for heading date variation. **(B)** Senescence variation recorded for a segregating RIL population (gray, *n* = 75), staygreen parent (green), non-staygreen parent (orange) **(B)**; mean visual senescence score (*n* ≥ 2), Church Farm, Bawburgh, Norwich, 2018.

Monocarpic senescence is the final stage in wheat development, during which 80% of leaf nitrogen and phosphorus are re-assimilated into the developing grain (Buchanan-Wollaston, 2007). Senescence is subject to strong environmental and genetic regulation, and prior to visual yellowing and chlorosis up to 50% of leaf chlorophyll may be lost (Buchanan-Wollaston et al., 2005; Borrill et al., 2019). Despite this, senescence progression is typically monitored through recording changes in leaf greenness or chlorophyll content over time, either at the individual flag leaf or canopy level (Figure 1 and Table 1; Pask et al., 2012; Shrestha et al., 2012). With reference to Wheat Initiative and CIMMYT crop ontology (Shrestha et al., 2012), “Flag Leaf Senescence” (CO_321:0000194) is commonly assessed using a scale from 0 (0% senescence) to 10 (100% senescence) (CO_321:0000382; Pask et al., 2012). Alternatively, Normalized Differential Vegetation Index (NDVI; CO_321:0000301) or Green NDVI (GNVDI; CO_321:0000961) can be measured using spectral reflectance, where the change in canopy greenness or photosynthetic size provides a more objective measure of senescence (Table 1).

**TABLE 1:** Reviewing methods used to score, quantify, and describe leaf senescence progression. Senescence phenotyping concerns monitoring changes in greenness over time for which multiple methods can be used. To characterize, compare, and quantify senescence phenotypes, time course senescence data can be transformed into a series of parameters (Figure 2) using a variety of approaches.

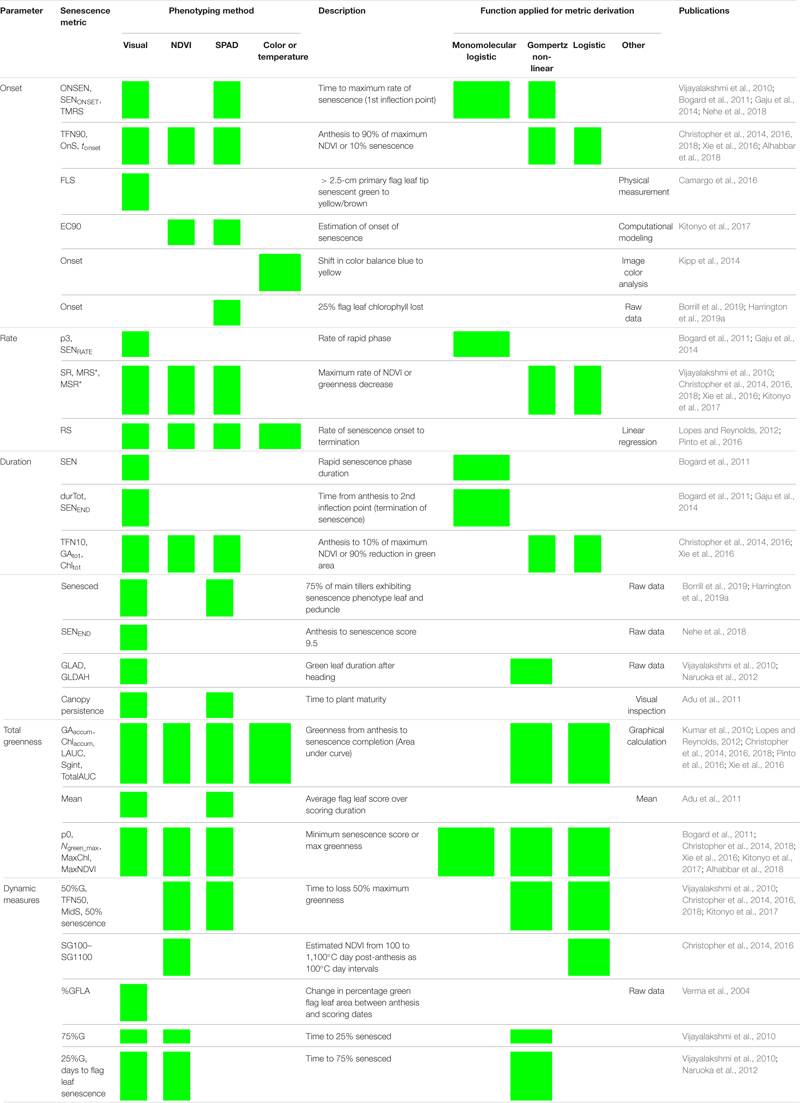

*Shading indicates the method of phenotyping and metric calculation.*

Crop ontology defines the “staygreen” trait (CO_321:0000059) as the “ability of the plant to remain/maintain green leaves, stems, and spikes at the time of senescing” (Shrestha et al., 2012). However, only functional staygreen phenotypes are considered useful due to their association with prolonged or enhanced photosynthetic activity, compared to cosmetic types in which chlorophyll catabolism is impaired (Gregersen et al., 2013; Thomas and Ougham, 2014). Unfortunately, senescence phenotyping efforts often concentrate on recording changes in leaf, or canopy, color and not the accompanying developmental and physiological changes, potentially favoring identification of cosmetic staygreens as opposed to useful types.

A direct correlation between green canopy and grain fill duration is frequently assumed, which experiments by Wiegand and Cuellar (1981) and Gelang et al. (2000) confirm. However, when studying senescence this relationship is rarely explicitly tested, although Pinto et al. (2016) report significant correlations between residual greenness at maturity, determined using NDVI, with grain fill duration, *r* = −0.35 to 0.58, *P* < 0.0001. Loss of glume color and peduncle ripening are also associated with changes in grain development, coinciding with GS87, the timepoint at which dry grain weight is maximal (Pask et al., 2012). Ear photosynthesis contributes to between 40 and 80% of grain carbohydrate (Zhou et al., 2016), with ears supplying 1.87 times more nitrogen compared to flag leaves (Barraclough et al., 2014). Additionally, peduncle senescence has important implications regarding the delivery of flag leaf-derived photosynthates. Peduncles act as conduits and stores for transient starch and sugars, facilitating their remobilization into the grain, while carbohydrates within peduncle tissue help maintain hydraulic conductance (Raven and Griffiths, 2015). If peduncles senesce in advance of leaves then the photosynthates associated are unable to reach the grain. Together, this illustrates the need for spike and peduncle phenotyping, alongside recording of grain filling dynamics when studying senescence, for which Table 2 lists the methods adopted.

**TABLE 2:** Senescence scoring efforts should not be limited to leaves. Senescence is a whole-plant process involving remobilization of resources into grain, for which studying multiple organs has been used to aid our understanding.

Component	Phenotype	Scoring	Metric	Publications
Grain filling	Grain weight and moisture content	Developmental time course, recording grain weight and moisture content	Genotypic pairwise comparison	Uauy et al., 2006b; Borrill et al., 2019
			
			Logistic growth curve modeling of grain fill dynamics: GFD, grain fill duration Gfr, grain filling rate; maximum, rapid, late, average Tmax, time of Mgfr Mwc, maximum water content Tmwc, time of Mwc WAR, water absorption rate Wlr, water loss rate	Xie et al., 2016
		
		Yield, final grain weight, plant maturity.	GFR, grain filling rate = yield / (days to maturity–days to heading) Gdecay % NDVIg decline during initial grain fill CTgf, canopy temperature during grain fill	Lopes and Reynolds, 2012; Pinto et al., 2016

Peduncle	Color transition from green to yellow	Plot level: % of yellow peduncles	1 measurement (30 daa)	Uauy et al., 2006b
			
			50% yellow peduncles (GS89)	Pask et al., 2012; Nehe et al., 2018
			
			“Senesced” 75% plants with totally yellow peduncles	Harrington et al., 2019a, b
		
		Time course: 2-day intervals from anthesis	Days from anthesis to 100% yellow	Uauy et al., 2006b; Borrill et al., 2019; Harrington et al., 2019a
		
		Peduncle chlorophyll content	Genotypic pairwise comparison (33 and 49 daa)	Harrington et al., 2019b

Spike	Color transition, weight, and moisture content	Total spike weight and moisture content (mid to late grain filling)	1 measurement (30 daa)	Uauy et al., 2006b
			
			Genotypic pairwise comparison during late grain fill	Avni et al., 2014
		
		Difference in leaf and spike greenness scored using a 0–10 scale at GS87	Staygreen: <3–6 Moderate staygreen: >2–<3 Moderately non-SG: >1–<2 Non-SG: 0–<1	Kumar et al., 2010
		
		50% spikes bleached	PM (plant maturity)	Lopes and Reynolds, 2012; Pinto et al., 2016
		
		Complete spike senescence	Days from anthesis	Uauy et al., 2006b

**FIGURE 2:**
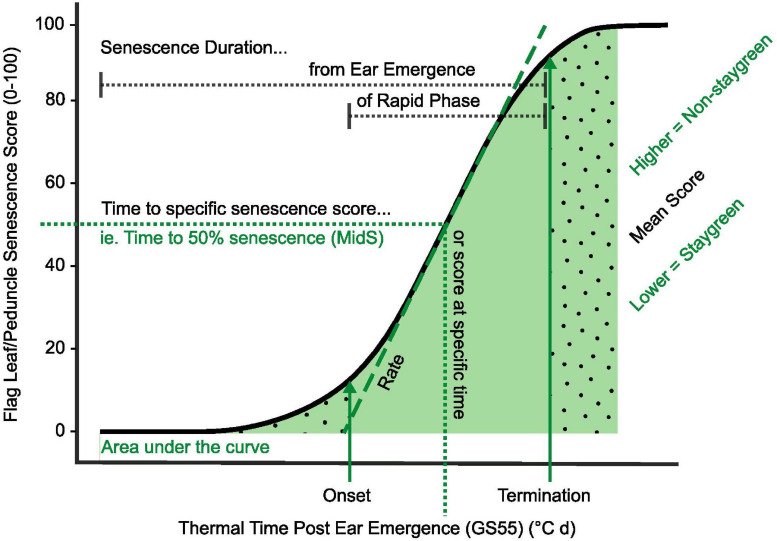
Key parameters used in quantification and characterization of senescence variation. Metrics relating to onset, rate, and duration of senescence, alongside total greenness (area under the curve) and dynamic measures (including MidS), can be calculated from time course data (Table 2). Onset of senescence marks the transition between the initial lag phase (dotted fill, right) and rapid phase of senescence (solid fill, center). As senescence nears completion, the senescence rate decreases, resulting in a final lag phase (dotted fill, left). To compare senescence progression of different lines, time to different senescence scores (MidS), an overall mean, or total greenness can be calculated.

Recently, staygreen traits have received renewed interest due to their potential ability to increase yield and stress tolerance (Gregersen et al., 2013; Jagadish et al., 2015). For example, multiple studies report the indirect selection of staygreen traits over the previous 50 years as helping sustain grain number improvement (Adu et al., 2011; Kitonyo et al., 2017; Voss-Fels et al., 2019). Modeling of wheat ideotypes using 2050 climate change predictions weights staygreen traits highly, estimating associated yield benefits of 28–37% and 10–23% for Spanish, and Central and Eastern European growing regions, respectively (Senapati et al., 2019). Under stress, Chapman et al. (2020) reported a positive relationship between delayed senescence and grain weight improvement of *NAM-1* ethyl methane sulphonate (EMS) mutants, which could relate to elevated ABA levels enhancing carbon remobilization (Distelfeld et al., 2014). However, not all staygreen phenotypes are the same, with senescence dynamics a product of the differences in onset, rate or duration, or initial chlorophyll content (Figure 2). Xie et al. (2016) hypothesize that a delay in onset, coupled with a rapid rate, of senescence maximizes remobilization efficiency, reporting rapid grain fill rate and TGW as correlated, *r* = 0.63–0.77, *P* < 0.01. Conversely, Gelang et al. (2000) report senescence duration as the greatest contributing factor to grain fill, with traits highly associated, *R*^2^ = 0.989. In combination, elucidating the optimal combination of senescence dynamics under differing conditions is required to identify their associated target breeding environments and stimulate staygreen trait adoption.

For studies investigating a limited number of lines, plotting and visual comparison of senescence time course data may be sufficient to identify and characterize senescence variation. However, when assessing senescence of segregating recombinant inbred line (RIL) populations or a diversity set, visual discrimination of individual senescence profiles is challenging (Figure 1). Transformation of senescence curves into a series of well-defined metrics aids in the characterization of individual lines, allows senescence dynamics to be described, and permits the performance of quantitative analysis, including quantitative trait locus (QTL) mapping. Unfortunately, multiple studies apply their own methods to derive senescence metrics, leading to varying definitions of apparently similar terms (Table 1). In the absence of consistent senescence phenotyping approaches results from different studies cannot be directly compared, preventing the interpretation of significant genotypic and environmental variation (Verma et al., 2004; Pask et al., 2012; Christopher et al., 2014).

Recognizing the disparity in senescence phenotyping methods present in the literature, we reviewed, used, and developed a range of methods that successfully capture senescence variation observed among two segregating RIL populations. Although duration and onset of senescence are the metrics mostly used to describe senescence dynamics (Table 1), these can fail to capture process dynamism and source-to-sink relationships. Simultaneous scoring of flag leaf and peduncle senescence, alongside monitoring changes in grain development, improved our understanding of senescence processes at a whole-plant level. We also identified the need for singular metrics capable of discriminating between staygreen and non-staygreen types, preferably in the absence of time course phenotyping, to increase efficiency of in-field phenotyping and selection. Here we compile resources we referred to when scoring senescence under field conditions, and provide our own insights following successful mapping of *NAM-1* homeologs, which are known senescence regulators (Chapman et al., 2020). Here we aim to equip researchers and breeders alike with appropriate knowledge to guide future phenotyping strategies, support trait genetic mapping and selection, and help inform weighting of senescence traits in breeding, genomic selection, or other applications.

## Materials and Methods

### Plant Material

Phenotypic data relates to two *Triticum aestivum* cv. Paragon EMS staygreen mutants and associated RIL populations. “Staygreen A” and “Staygreen B” refers to mutant lines 1189a and 2316b, identified as encoding missense mutations in known senescence regulators *NAM-A1* (T159I) and *NAM-D1* (G151), respectively (Chapman et al., 2020). “Non-staygreen” refers to the parental *T. aestivum* cv. Paragon, and Staygreen A and B were selected based on their differential staygreen phenotypes, agronomic potential, and similarity in heading date. To develop segregating RIL populations, mutants were crossed to cv. Paragon and F_4_ populations developed through SSD, *n* ≥ 85.

### Field Experiments

Phenotyping of F_4_ RIL populations was conducted under field conditions between 2016 and 2018. Experiments were performed at Church Farm, Bawburgh, Norwich (52°38′ N, 1°10′ E), JIC, as described previously (Chapman et al., 2020). In brief, 36 to ≥ 75 RILs were sown per population per year as unreplicated 1 m^2^ plots (2016) or replicated 6 m^2^ plots (*n* = 3, 2017; *n* = 2, 2018). Seeds were sown on 26/10/2016, 26/10/2017, and 12/10/2018 at a rate of 250–300 seeds m^–2^. Replicated experiments followed a randomized complete block design, with control plots of cv. Paragon, Soissons, 1189a, and 2316b mutant lines randomly sown throughout. Seed used for experiments was produced during the multiplication of RIL populations in 2015 or resulted from the previous year. Soil at Church Farm is described as sandy loam overlying alluvial clay. Supplemental irrigation was applied in 2017; otherwise, trials were naturally rainfed. Fertilizer was applied over three occasions from late February to the end of April, totalling 214 kg N ha^–1^ and 62 kg SO_3_ ha^–1^ in 2017 and 228.5 kg N ha^–1^ and 62 kg SO_3_ ha^1^ in 2018. The plots received standard fungicide and herbicide treatment. Rainfall and temperature data corresponding to each field season are supplied in Supplementary Figure 1.

### Phenotypic Assessment

**FIGURE 3:**
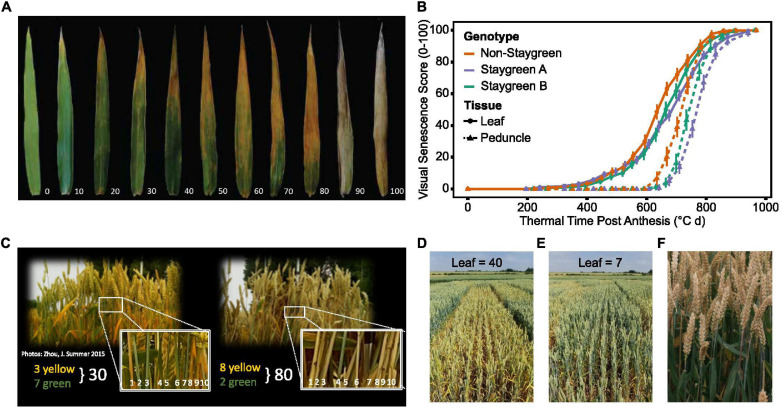
Simultaneous scoring of flag leaf, peduncle, and ear senescence allows the whole plant nature of senescence to be considered. Compared to flag leaf senescence **(A)**, scoring of peduncle senescence **(C)** is less subjective, and respective senescence profiles were found to reinforce one another **(B)**. Senescence of multiple plants was visually assessed and scored using a 0–100 scale **(A,C)** at the plot level **(D,E)**, taking care to avoid edge effects or diseased plants. When observed, asynchronous senescence phenotypes **(F)** were recorded and may indicate impaired remobilization efficiency.

Ear emergence (GS55) was recorded as the point when 50% of ears emerged halfway from the flag leaf across the plot (Zadoks et al., 1974). Visual scoring of flag leaf and peduncle senescence was conducted at the same time using a 0–100 scale (intervals of 5) (Figure 3) every 2–3 days from ear emergence until maturity.

Flag leaf senescence was scored as the proportion of flag leaf yellowing with a score of 5 indicating leaf tip necrosis, and 100 complete senescence (Figure 3A). To avoid edge effects, flag leaves of multiple plants within plot centers were assessed together to derive an overall plot score (Figures 3D,E). Instances of plot heterogeneity resulting from disease, soil gradients, or damage were also recorded and subsequently referred to for the purpose of outlier detection. To reduce systematic error, plots were scored in the same orientation and direction on each visit, with scoring in direct sunlight avoided due to increased difficulty of identifying plot differences.

Peduncle senescence was scored as the percentage of plants for which the top 5 cm of peduncle tissue had transitioned from green to yellow. Compared to flag leaves, peduncles senesce evenly along their length, and the phase of rapid senescence is shorter (Figure 3B). This rapid color transition enables peduncles to be scored as either completely green or completely yellow, increasing objectivity of assessment. To accurately determine genuine differences between plots, 3–4 batches of 10 tillers were assessed and percentage yellow derived (Figure 3C).

To identify any potential association between grain filling and senescence phenotypes, grain maturity of RIL populations was scored according to the Zadoks scale with reference to the Wheat Growth Guide (Zadoks et al., 1974; AHDB, 2018). Two to three immature grains, from two plants per plot, were subject to thumbnail impressions or squashed between finger and thumb to determine the developmental stage, with observations recorded using a 1–4 scale (hard to soft) or Zadoks growth stages, GS79–GS93 (milky dough to ripe, grain loosening in the daytime) (AHDB, 2018). When observed, differences in flag leaf and ear senescence were recorded, which, in extreme cases, manifested themselves as “green leaf, ripe ear” phenotypes (Figure 3F). Through recording changes in flag leaf and peduncle greenness, alongside grain and spike maturity, one can understand the whole-plant nature of senescence, providing insights into resource remobilization and source-to-sink relationships. Table 2 lists senescence phenotyping approaches applied by other studies.

### Derivation of Senescence Metrics

To quantify and interpret senescence dynamics multiple senescence metrics can be derived (Figure 2). Flag leaf and peduncle senescence scores were plotted against thermal time post-ear emergence GS55 (°C day) to standardize for heading variation and associated differences in temperature exposed. Mean daily temperature was calculated using daily minimum and maximum temperatures and summated over time in days, with GS55 corresponding to 0°C days. The metrics used to describe senescence patterns fall into five categories corresponding to onset, rate, duration, total greenness, or a dynamic measurement of senescence; however definitions of these terms alongside their method of calculation vary (Table 1).

The sigmoidal progression of senescence facilitates curve modeling, whereupon senescence is divided into three phases: an initial lag phase, a rapidly senescing phase, and a final lag maturation phase (Table 1). To calculate the rate, onset, and duration of senescence, we initially applied the model described by Bogard et al. (2011) and Gaju et al. (2011) to our data. A comparison of raw and modeled curves found these calculated metrics were of limited use, identifying a tendency of the model to overfit the data, while infrequent scoring led to inaccurate calculation of inflection points.

**FIGURE 4:**
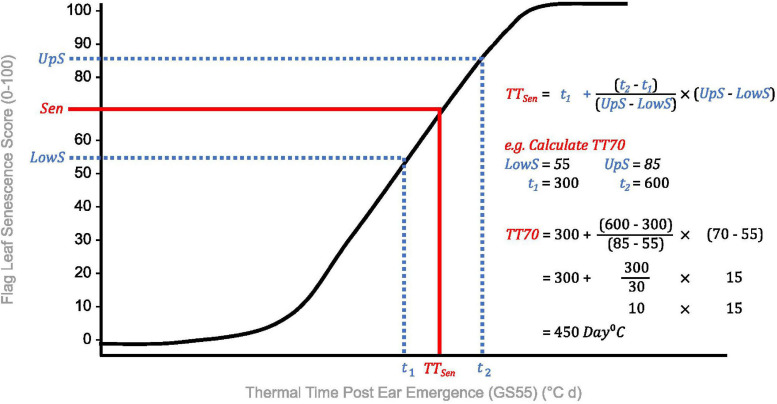
Calculation of thermal time to flag leaf senescence score of 70 (TT70), a dynamic senescence metric. Senescence scoring dates are converted into thermal time from ear emergence (°C d) to standardize for heading date variation. A “target” flag leaf senescence score is specified (Sen), and time points for scores below (*t*_1_, LowS) and above (*t*_2_, UpS) this are identified. Assuming senescence progresses linearly, time elapsed between flag leaf senescence scores is divided proportionately to estimate the time to the target score (TT_Sen_).

Due to problems encountered when curve modeling, we reviewed the definitions of commonly calculated metrics (Table 1) to inform derivation of senescence metrics from raw data (Table 3). Concordant with Christopher et al. (2014, 2016, 2018), Xie et al. (2016), Kitonyo et al. (2017), and Alhabbar et al. (2018), we define the onset of senescence as the “start of rapid senescence phase (flag leaf senescence score of ∼10–15)” (Table 3). Similar to Christopher et al. (2014, 2016), Xie et al. (2016) and Nehe et al. (2018), we define termination of flag leaf or peduncle senescence as the “time at which maximum senescence score (>90) is first recorded” (Table 3). Senescence duration was calculated from both ear emergence and onset of senescence (Figure 2), with the latter providing an indirect measure of senescence rate (Table 3). To capture senescence dynamism, we derived times to different flag leaf senescence scores (Table 3), similar to metrics MidS (midpoint of senescence) and 75%G utilized by Vijayalakshmi et al. (2010) and others (Table 1). Assuming that senescence progresses linearly, time points corresponding to scores above and below the “target” senescence score, i.e., 70, are identified with time divided proportionately to estimate time elapsed (Figure 4). Calculating the mean senescence score over the scoring period provides an assessment of overall greenness (Nehe et al., 2018), and was calculated separately for both flag leaf senescence (MeanLeaf) and peduncle senescence (MeanPed) (Table 3).

**TABLE 3:** Senescence metrics derived for quantification and qualification of time course senescence profiles.

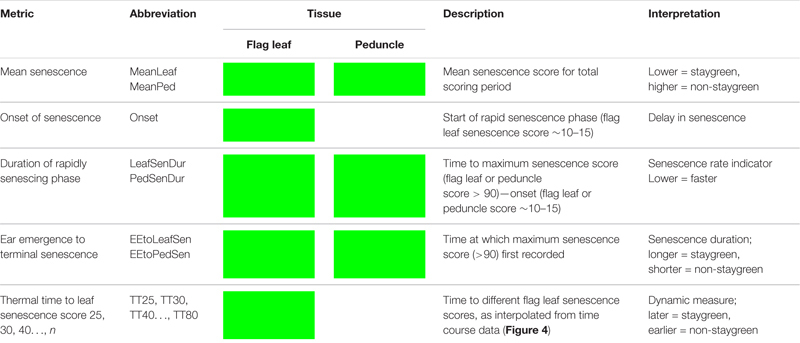

*Shading indicates the tissue phenotyped.*

### Data Analysis

Data analysis was performed using R (version 3.5.2) (R Core Team, 2018) within R Studio (R Studio team., 2015), and data manipulated using the packages “data.table” (Dowle et al., 2019), “dplyr” (Wickham et al., 2018b), “plyr” (Wickham, 2015), and “tidyr” (Wickham et al., 2019). Senescence metrics were derived from raw senescence data in absence of spatial correction with means calculated per line when replicated. To assess heritability and accuracy of senescence scoring Spearman’s rank correlations were calculated and results visualized using “ggpubr” (Kassambara, 2018). To illustrate the discriminatory power of senescence metrics, phenotype × genotype plots were constructed using the package “r/qtl” (Arends et al., 2010), with other graphs produced using the package “ggplot2” (Wickham et al., 2018a). To determine the significance of phenotypic differences linear mixed modeling was performed using the packages “lme4” (Bates et al., 2019) and “lmerTest” (Kuznetsova et al., 2017), with replicate, row, column, and *NAM-1* genotype treated as fixed effects, and RILs per population as random. Tukey *post hoc* tests were performed using the package “lsmeans” (Lenth, 2018).

## Results

### The Parallel Progression of Peduncle Senescence

Few publications report the use of peduncle senescence phenotyping when studying senescence regulation (Table 2). In 2017 and 2018 we conducted time course senescence scoring of flag leaf and peduncle tissue (Figure 3). We identified that peduncle senescence is initiated after flag leaf senescence, with senescence profiles found to reinforce one another, aiding differentiation of lines (Figure 3B; Chapman et al., 2020). Compared to flag leaf senescence, peduncle senescence occurs over a shorter time period, and individual scores demonstrate less variation as indicated by their comparatively smaller error bars (Figure 3B).

### Methods for Accurate Capture of Senescence Variation

Between 2016 and 2018, RILs segregating for two independent senescence traits were phenotyped under field conditions. Plotting senescence data against thermal time enabled calculation of senescence metrics, illustrating the effect of environment on senescence regulation. For example, between 2016 and 2018, ear emergence to terminal flag leaf senescence (EEtoLeafSen) scores ranged from 825 (95% CI 792, 858) to 1,156 (95% CI 1,090, 1,220) °C d for the “non-staygreen” parent; a difference of 331°C d. Differences in senescence duration reflect variation in mean daily temperature, with senescence progressing most rapidly and terminating earliest in 2018 due to elevated mean daily temperatures and reduced rainfall (Supplementary Figure 1).

To determine the stability of senescence phenotypes, and accuracy of phenotyping methods, year-pairwise Spearman’s rank correlations were calculated for a range of senescence metrics. We found that the magnitude and significance of between-year phenotypic correlations relates to the penetrance and stability of parental senescence phenotypes. For example, Staygreen A RILs segregate for a relatively extreme staygreen phenotype (Figure 3B), and year-pairwise phenotypic correlations ranged from *r* = 0.39 to 0.91, *P* ≤ 0.02 (Supplementary Table 1). Conversely, Staygreen B RILs segregate for a milder staygreen phenotype, and year-pairwise phenotypic correlations were lower, ranging from *r* = 0.3 to 0.68, *P* ≤ 0.03 (Supplementary Table 1). Correlations were significant for all 3 year-pairs for between two and nine metrics, *P* ≤ 0.03, indicating trait heritability, environmental stability, and accurate phenotyping of RILs (Supplementary Table 1). Insights from such analysis can direct in-field phenotyping approaches, informing which senescence metrics to prioritize when conducting phenotypic selection and forward genetic screens.

**FIGURE 5:**
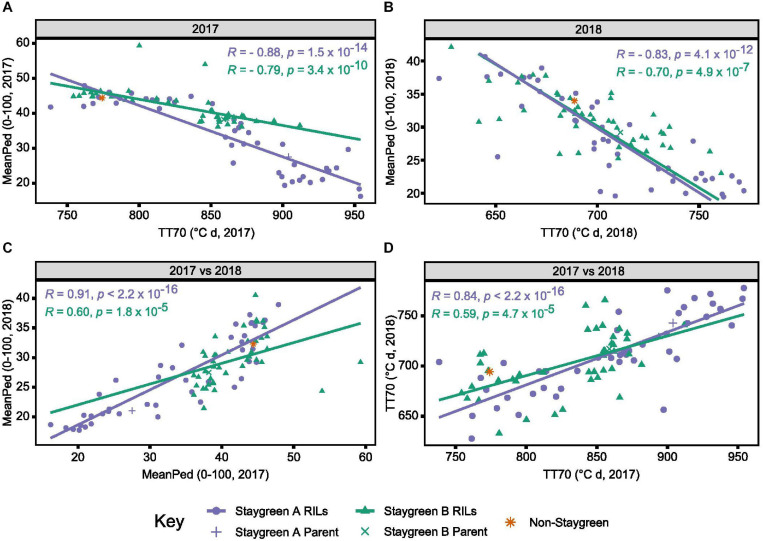
Environmental stability and concordance of metrics TT70 and MeanPed aids in the discrimination of staygreen and non-staygreen types. Through comparing TT70 and MeanPed values recorded in 2017 **(A)** and 2018 **(B)**, one can distinguish senescence variation, whereby “staygreen” and “non-staygreen” lines cluster in the bottom-right and top-left corners, respectively. Between-year Spearman’s rank correlations for metrics MeanPed **(C)** and TT70 **(D)** range from *R* = 0.59 to 0.91, *P* < 0.0001, with the metrics themselves negatively correlated, *R* = 0.7 to 0.88, *P* < 0.0001 **(A,B)**. Spearman’s rank correlations calculated for the 36–42 RILs per population grown at Church Farm, Bawburgh, Norwich (JIC) between 2017 and 2018, plotting mean value per line, *n* ≥ 2. Staygreen A parent and RILs (purple), staygreen B parent and RILs (green), and recurrent parent (orange).

As time course phenotyping is time consuming and laborious, we identified the need for a single senescence metric capable of discriminating senescence types. Magnitude and significance of year-pairwise correlations calculated for the same metric enable environmental stability and metric performance to be assessed. Inspection of year-pairwise correlations identified metric TT70 as a potential candidate, with correlations of *r* = 0.78–0.84 and *r* = 0.37–0.59, *P* ≤ 0.02, reported for Staygreen A and Staygreen B RILs, respectively (Figure 5D and Supplementary Table 1). Alternatively, peduncle senescence-derived metrics may better discriminate senescence phenotypes. Compared to metric TT70, year-pairwise correlations for MeanPed (mean peduncle senescence) scores were greater, ranging from *r* = 0.62 (Staygreen B) to 0.91 (Staygreen A), *P* ≤ 1.8 × 10^–5^ (Figure 5C), indicating greater environmental stability.

### Coupling MeanPed and TT70 Scores Aids Selection and Discrimination of Senescence Types

Calculation of year-pairwise phenotypic correlations illustrates that our approach to senescence scoring and quantification is robust, but does it aid in the discrimination of senescence variation among lines? Correlation plots displaying mean TT70 or MeanPed scores recorded for individual RILs reveals their tendency to cluster into senescence types. For metric TT70, RILs clustered toward the bottom-left corner are considered “non-staygreen,” with those clustered toward the top-right “staygreen” due to taking longer to senesce (Figure 5D). Conversely, lower MeanPed scores indicate greater retention of green peduncle tissue, with RILs clustered toward the bottom-left considered “staygreen” (Figure 5C).

The degree of separation between “non-staygreen” and “staygreen” clusters relates to the extremity of senescence phenotype. For example, the mean difference in TT70 scores between lines Staygreen A and B compared to the parental non-staygreen line are 85.3 ± 34.4 and 63.0 ± 27.8°C d, respectively, with contrasting Staygreen A RILs clustering further apart (Figure 5D). Greater robusticity of peduncle-derived senescence scores contributes to tighter clustering of RILs contrasting for senescence phenotypes (Figure 5C), although metric TT70 can distinguish smaller variations between RILs, due to a greater range of recorded values (Figure 5D). In combination, metrics TT70 and MeanPed can be used to accurately discriminate senescence types, particularly in the absence of multi-year phenotyping data as we found the metrics to be highly correlated, *r* = −0.7 to −0.88, *P* ≤ 4.9 × 10^–7^ (Figures 5A,B).

**FIGURE 6:**
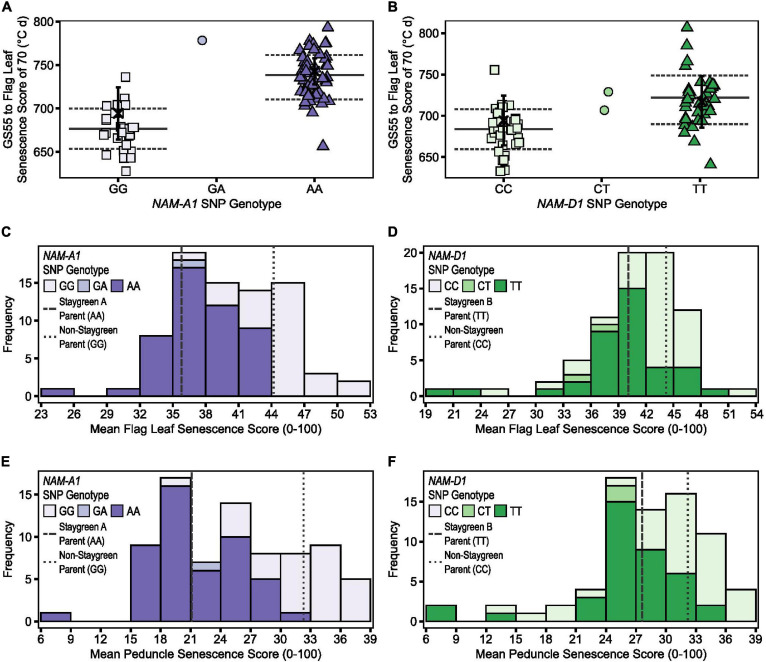
Illustrating the differential power of metrics TT70, MeanLeaf, and MeanPed in discriminating senescence variation. Phenotypic differences were significant between homozygous RILs contrasting for mutations in *NAM-A1* (Staygreen A) **(A,C,E)** and *NAM-D1* (staygreen B) **(B,D,F)** according to TT70 **(A,B)**, MeanLeaf **(C,D)**, and MeanPed **(E,F)** scores, *P* ≤ 0.003. Metric MeanPed proved more informative when classifying senescence types compared to MeanLeaf due to the greater range and spread of scores. Phenotypic differences between parents were not significant, *P* > 0.05. **(A,B)** Scatterplot of TT70 scores against *NAM-1* genotype; genotypic group mean (solid line) ± SD (dotted line), parental mean (black cross) ± SD. **(C–F)** Bars display mean MeanLeaf and MeanPed score recorded for two RIL populations, *n* ≥ 75 (two replicates) segregating for mutations in *NAM-A1* **(C,E)** or *NAM-D1* **(D,F)**; allelic combinations, GG/CC = homozygous non-staygreen, AA/TT = homozygous Staygreen A/Staygreen B, GA/CT = heterozygous. Lines represent parental means (*n* > 5); staygreen parents (dashed), non-staygreen parent (dotted). Trial grown at Church Farm, Bawburgh, Norwich (JIC), 2018.

Proof of the utility of TT70 and MeanPed scores in discriminating senescence variation are the results of mapping by bulk segregant analysis conducted for Staygreen A and Staygreen B (Chapman et al., 2020). While different senescence metrics may better capture the range of senescence variation in different years, assessment of TT70 and MeanPed scores consistently identified the same RILs for which senescence was delayed, facilitating the selection of phenotypically contrasting bulks. Compared to metric MeanLeaf, the distribution of MeanPed scores recorded for Staygreen A and B RILs was flatter, indicating that peduncle-derived senescence scores could better discern senescence variation (Figure 6).

However, although differences in flag leaf and peduncle senescence profiles of Staygreen A and B were typically significant relative to the non-staygreen line, *P* < 0.0001 to *P* = 0.11 (Chapman et al., 2020), differences in TT70 and MeanLeaf scores were not significant in 2018, *P* > 0.05, but were significant in 2016 and 2017, *P* < 0.05. Similarly, differences in MeanPed scores recorded for Staygreen A and the non-staygreen parent were significant only in 2017, *P* < 0.01, whilst differences observed for Staygreen B were not, *P* > 0.05. Therefore, when differences in a senescence metric are not significant between lines under investigation, one recommends their comparative assessment, just as we performed when classifying senescence variation among RILs during mapping of Staygreen A and B (Chapman et al., 2020). This approach worked successfully as, following trait mapping, differences in TT70 and MeanPed scores recorded for homozygous RILs contrasting for mutations in *NAM-A1* or *NAM-D1* (Staygreen A and B, respectively) were significant in all 3 years, *P* < 0.05 (Figure 6).

### Determining the Association Between Delayed Senescence and Grain Maturity

Previously, grain filling experiments conducted for Staygreen A and Staygreen B found grain moisture content remained elevated during the final 10–15 days of grain filling relative to their non-staygreen parent, *P* < 0.05 (pairwise Tukey *post hoc* test; Chapman et al., 2020). The number of time points for which grain moisture content of Staygreen A and Staygreen B were significantly greater compared to their common non-staygreen parent corresponded to the observed differences in onset of senescence, indicating a positive trait association (Chapman et al., 2020). Variation in grain development was detected amongst RIL subsets segregating for senescence traits grown in 2016 and 2017 (data not shown). However, because Staygreen A and B were produced through EMS mutagenesis, involvement of background mutations could not be discounted. Differences in grain moisture content were not attributable to phenological differences, with heading date variation limited to 1–2 days for each population.

**FIGURE 7:**
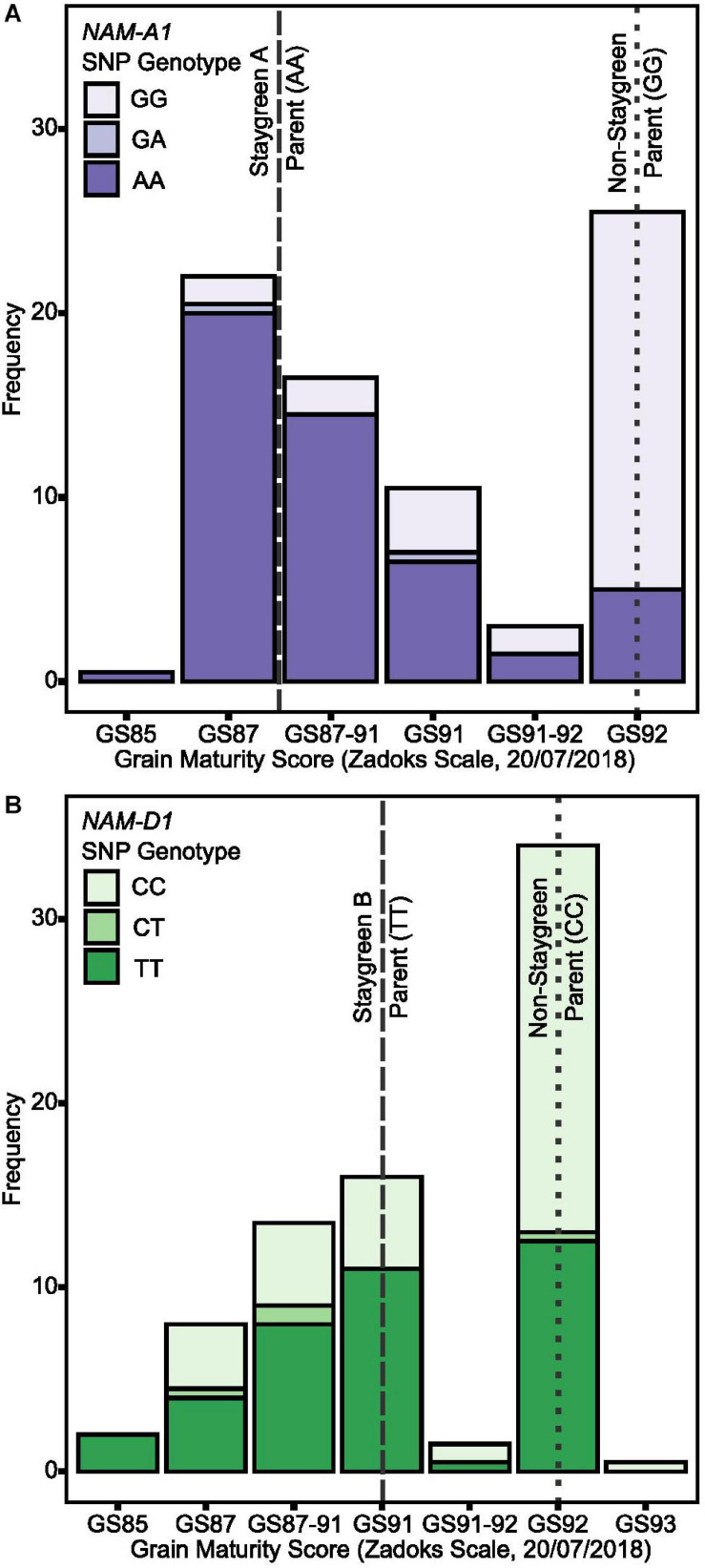
Staygreen phenotypes are positively associated with delayed grain maturation. Grain maturation of RIL populations segregating for senescence traits was assessed when significant differences in grain moisture content between parental lines (Staygreen A vs. non-staygreen parent and Staygreen B vs. non-staygreen parent) were previously observed. Grain maturation of RILs homozygous for mutations in *NAM-A1* (Staygreen A) **(A)** and *NAM-D1* (Staygreen B) **(B)** was delayed, with scores starting from GS85 onwards, compared to the predominant score of GS92 for RILs homozygous for the non-staygreen allele. Grain scored according to the Zadoks scale (Zadoks et al., 1974), assisted by “The Wheat Growth Guide” (AHDB, 2018). Bar charts represent RIL populations, *n* ≥ 75 (two replicates) segregating for a mutation *NAM-A1* (Staygreen A) **(A)** or *NAM-D1* (Staygreen B) **(B)**; allelic combinations, GG/CC = homozygous non-staygreen, AA/TT = homozygous Staygreen A/Staygreen B, GA/CT = heterozygous. Lines represent parental means (*n* ≥ 3 per replicate); staygreen parents (dashed), non-staygreen parent (dotted).

To conduct grain filling experiments for entire RIL populations would have been unfeasible; however, thumbnail impressions are routinely performed to assess grain development and maturity (Zadoks et al., 1974; AHDB, 2018). On July 20, 2018, grain filling experiments found grain moisture content of Staygreen A and B to be significantly elevated compared to the non-staygreen parent, *P* < 0.001 (pairwise Tukey *post hoc* test; Chapman et al., 2020). Thumbnail-based maturity scores ranged from GS87-91 for the Staygreen A parent (*n* = 4) and Staygreen B parent (*n* = 3) and GS92 for the non-staygreen parent (*n* = 7) (Figure 7). Grain maturity of some plots was recorded as being between two growth stages, GS87-91 and GS91-92, attributable to variation between main and secondary tillers.

Grain maturity scores recorded for RIL populations (*n* ≥ 75) ranged from GS85 (soft dough) to GS93 (grain loosening in daytime) (AHDB, 2018) which, when plotted against *NAM-1* SNP composition, confirm that senescence and grain filling traits are associated (Figure 7). On July 20, 2018, a grain maturity score of GS92 was recorded for 71 and 59% of RILs homozygous for the “non-staygreen” *NAM-A1* (GG) or *NAM-D1* (CC) allele, respectively (Figure 7). In contrast, 73% of RILs homozygous for the Staygreen A *NAM-A1* allele (AA) recorded a grain maturity score of GS87-91 or below (Figure 7A). Differences in grain maturity were subtler for Staygreen B, and scores of GS85 to GS87-91 and GS91 were recorded for 37 and 29% of RILs homozygous for the *NAM-D1* allele (TT), respectively (Figure 7B). Previous grain filling experiments identified the relative timing of differences in grain development for Staygreen A and B (Chapman et al., 2020), for which thumbnail impression-based scoring can quickly and accurately capture such differences based on these results. Assessing visual leaf and peduncle senescence together with grain maturation could provide a method to separate cosmetic and functional staygreen phenotypes. Furthermore, through recording visual flag leaf and peduncle senescence scores when grain maturity is reached, one can identify if resources are being efficiently remobilized into the grain (Figure 3F).

## Discussion

### Perks of Peduncle Senescence Scoring

When mapping the *GPC-B1* locus, Uauy et al. (2006a) reported changes in peduncle color as being linked. This resulted in the identification of the NAC transcription factor and senescence regulator *NAM-B1* (Uauy et al., 2006b). Subsequent reverse genetic studies investigating the role of *NAM-1* homeologs and paralogs in senescence regulation independently confirm the utility of peduncle phenotyping, with these accurately capturing senescence variation (Cantu et al., 2011; Avni et al., 2014; Pearce et al., 2014; Borrill et al., 2019; Harrington et al., 2019a). Compared to flag leaf senescence, peduncle senescence is initiated later and progresses rapidly, with visual yellowing of peduncles first observed when flag leaf senescence scores approach ∼50 (Figure 3B). Unlike flag leaves, peduncles senesce evenly along their length making plot-level assessment objective (Figure 3), with scores typically only confounded by barley yellow dwarf virus-associated anthocyanin production (Livingston et al., 1998).

In agreement with Borrill et al. (2019) and Harrington et al. (2019a, b), we report greater environmental stability of peduncle, as opposed to flag leaf, senescence phenotypes (Figures 5C,D). For example, when characterizing *Triticum turgidum* cv. Kronos *NAM-A1* mutants, Harrington et al. (2019a) reported peduncle senescence as consistently delayed under both field and glasshouse conditions, *P* < 0.05, whereas flag leaf senescence was not, *P* > 0.05 (glasshouse), *P* < 0.001 to *P* > 0.05 (field). Regarding our own lines, we found that peduncle and flag leaf senescence-derived metrics were highly correlated, *R* > 0.7, *P* < 0.001 (Figures 5A,B), illustrating that both phenotyping approaches accurately capture senescence variation. Together, increasing our reliance on peduncle senescence phenotyping may reduce the need for prolonged time course assessment, but to prevent this short window from being missed, flag leaf senescence requires monitoring.

### Quantitative to Qualitative: Selecting Senescence Types

**FIGURE 8:**
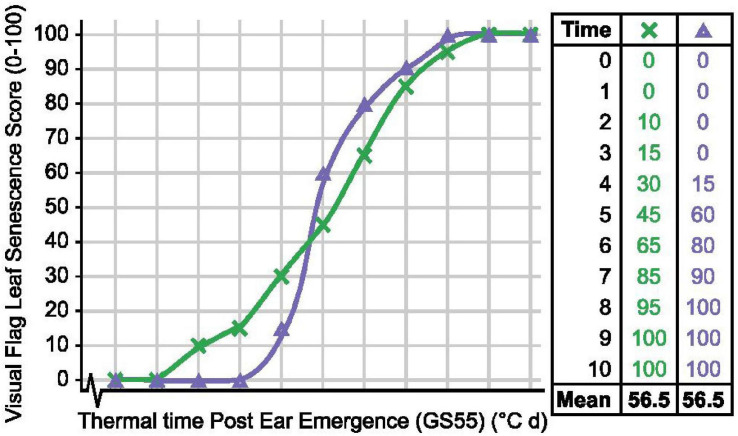
Mean senescence scores cannot always distinguish senescence variation. Senescence duration of line × (green) is prolonged, while onset of senescence is delayed and senescence rate is rapid for line Δ (purple), but their mean senescence scores are the same.

To quantify senescence, we initially calculated the mean flag leaf senescence score for the total scoring period (MeanLeaf). Comparing MeanLeaf scores of individual lines against their flag leaf senescence profiles revealed that the metric poorly captured senescence dynamism. Similarly, when evaluating 14 Australian wheat cultivars Kitonyo et al. (2017) reported cv. Heron as early but slow to senesce, and cv. Justica CL Plus as greener overall and rapidly senescing, but mean senescence scores may be similar (Figure 8). Using an alternative approach, Kitonyo et al. (2017) applied a logistic regression to time course NDVI measurements to quantify senescence, finding that maximum NDVI scores (near flowering) increased with year of cultivar release. In wheat, the use of the metric “mean senescence” is rare (Table 1), suggesting its limited utility, whereas in maize, Ziyomo et al. (2013) and Parajuli et al. (2018) successfully used mean scores to characterize stress responses of lines grown under different agronomic and cropping systems. Therefore, when quantifying senescence thought should be given to the specific pattern, or phase of senescence depicted, which may vary between systems.

In our study, the metric thermal time to flag leaf senescence score of 70 (TT70) proved most informative when discriminating senescence types. Unlike calculation of mean flag leaf senescence scores (LeafMean), estimation of the thermal time taken to reach different flag leaf senescence scores (TT25, TT30…) was less affected by pre-existing leaf damage resulting from nitrogen splash, leaf tip necrosis, or other damage. Pre-existing damage to flag leaves appears as “noise” during the early stages of senescence, however, following the onset of senescence, clear differences between lines emerge when flag leaf senescence scores range from 40 to 70 (Figure 1B). Depending on the extremity of the senescence phenotypes investigated, metric TT70 may be more informative than the duration of flag leaf senescence (EEtoLeafSen), which is more frequently used when recording senescence variation (Table 1). For example, in mild years plants may not reach terminal senescence, as we observed in 2016, preventing the calculation of EEtoLeafSen. In contrast, TT70 scores can be calculated prior to harvest, increasing the efficiency of phenotypic selection. On a cautionary note, while we identified metrics TT70 and MeanPed as most informative under our conditions, this may be germplasm dependent and not universally true. For example, Shi et al. (2016) observed that *T. aestivum* cv. Wenmai and cv. Lankaoaizao senesce non-sequentially, whereby flag leaves senesced prior to 2nd leaves, while the peduncles of *GPC-1* RNAi lines remained green (Cantu et al., 2011).

### Functional or Not? Understanding the Relationship Between Grain Filling and Leaf Senescence

A positive relationship between senescence and grain fill duration is often assumed, with grain fill duration and grain weight highly correlated, *r* = 0.77, *P* < 0.026 (Dias and Lidon, 2009). A study involving NDVI assessment of the *T. aestivum* Seri × Babax RIL population identified four QTLs commonly associated with senescence traits, grain fill duration, TGW, and yield located on chromosomes 1B, 2B, 2D, and 4B (Pinto et al., 2016). Meanwhile, QTL analysis of the *T. aestivum* cv. Spark × Rialto double-haploid population by Simmonds et al. (2014) identified a single QTL on chromosome 6A associated with green canopy duration, TGW, and yield. Using NILs contrasting for Rialto and Spark 6A alleles, yield and senescence traits co-segregated. The reported grain filling extension was associated with earlier flowering and delayed grain maturation, which occur 1 day earlier and ∼2 days later, for Rialto and Spark alleles respectively, not green canopy duration *per se* (Simmonds et al., 2014).

Differences in grain filling may be environmentally dependent. For example, under glasshouse conditions, no differences in grain maturation were recorded for *gpc-1* (*NAM-1*) mutants despite a delay in flag leaf senescence of 25 days (Simmonds et al., 2014; Borrill et al., 2015), whereas differences were observed among wheat lines carrying *NAM-A1* variants in dryland environments (Alhabbar et al., 2018). Conversely, the extended grain fill duration reported for Staygreen A and B, which encode mutations in *NAM-A1* and *NAM-D1*, respectively, was consistent over multiple years (Chapman et al., 2020), contributing to the observed heading-independent grain maturity differences (Figure 7).

The extended photosynthetic duration associated with functional staygreen phenotypes may provide additional resources for grain filling, supporting trait deployment in breeding. Xie et al. (2016) hypothesize that a delay in onset combined with a rapid rate of senescence maximizes grain weight and yield potential, especially as grain filling rate (growing °C days) and grain weight are correlated, *r* = 0.91, *P* = 0.002 (Dias and Lidon, 2009). Grain filling rate can be indirectly assessed through scoring senescence progression. For example, Xie et al. (2016) found rates of maximum chlorophyll loss and average grain fill to be correlated, *r* = 0.27–0.35, *P* < 0.01, however correlations between senescence metrics and grain fill duration were inconsistent, *r* = −0.4–0.4, *P* < 0.01 to *P >* 0.05. Combined, this emphasizes the need to score both time to terminal senescence and grain maturation, and would confirm the relationship assumed by Lopes and Reynolds (2012) and Pinto et al. (2016). We demonstrate that subjecting grain to thumbnail impressions can sufficiently characterize differences in grain maturation (Figure 7), providing a means of rapid assessment. If an objective assessment of grain maturation is required, we suggest recording the spike moisture content from ∼6 weeks after anthesis as conducted for *NAM-1* mutants (Avni et al., 2014).

Time to maximum senescence rate can coincide with maximal grain dimensions (Xie et al., 2015). Therefore, maintaining the synchronicity of senescence and grain filling processes is of concern when identifying or selecting staygreen traits. As photosynthesis terminates halfway through the rapid grain filling phase any delay could reduce the remobilization efficiency, as this marks the point when translocation and remobilization of stored reserves, fructose and sucrose occurs (Takahashi et al., 2001). Phenotyping of flag leaf and peduncle senescence, alongside grain maturity, could deliver insights into the process, and lines displaying “green leaf and ripe ear” phenotypes should be selected against (Figure 3F). Conversely, the grain fill extension reported for Staygreen A and B (Chapman et al., 2020) delayed grain maturation (Figure 7), which could disrupt harvest or adversely affect wheat quality through altering the deposition of triticin, glutenin, and gliadin storage proteins (Takahashi et al., 2001), and requires further investigation.

### Variable Trait Expression: Consider Target Environments

Staygreen traits are associated with conveying tolerance to heat, drought, and low nitrogen conditions (Gregersen et al., 2013; Jagadish et al., 2015), with trait expression strongly influenced by the environment. For example, of the 19 senescence QTLs mapped for the *T. aestivum* cv. Ventor × Karl92 RIL population, segregating for temperature responses, only three were environmentally stable (Vijayalakshmi et al., 2010). Individually, these three QTLs explained between 10 and 51% of senescence variation. Conversely, variation explained by the seven and nine senescence QTLs identified exclusively under high or optimal temperature conditions was lower, averaging *R*^2^ = 0.18 ± 0.11 and *R*^2^ = 0.14 ± 0.07, respectively (Vijayalakshmi et al., 2010). Therefore, screening for senescence traits under different environments may help identify potentially advantageous, stress-adaptive QTLs for use in breeding alongside major stable genetic regulators.

Water limitation also influences senescence. Estimated heritability of senescence traits recorded for the *T. aestivum* cv. Reeder × cv. Canan RIL population reduced from *H*^2^ = 0.78 under irrigated conditions to *H*^2^ = 0.51–0.81 when rainfed (Naruoka et al., 2012). Between-year weather variation can help identify putative epistatic interactions as documented during QTL mapping of the *T. aestivum* cv. Chirya-3 × Sonalika RIL population (Kumar et al., 2010). Kumar et al. (2010) identified an environmentally stable senescence QTL located on chromosome 1AS which, in combination with year-dependent QTLs located on 3BS (2005) and 7DS (2006), accounted for up to 38.7% of staygreen trait variation. While we identified both our staygreen traits as highly environmentally stable (Figure 5), we also recognize the influence of environment, as senescence was accelerated in 2017 and 2018 compared to 2016 due to increased temperature and water limitation (Supplementary Figure 1).

Altogether, these examples illustrate the need for repeated, preferably multi-environment, trialing to assess stress adaptivity or stability of senescence phenotypes. However, within breeding programs, lines are typically selected under high-input conditions, preventing phenotypic expression and selection of potential stress-adaptive staygreen phenotypes. From our experience, multi-environment, multi-year trials allowed us to identify potential penalties associated with the adoption of staygreen traits alongside appropriate target breeding environments. For example, although loss of glume color and grain ripening occurred ahead of flag leaf senescence in 2016 (Figure 3F), supporting non-adoption of staygreen traits (Jenner et al., 1991; Barraclough et al., 2014), results of continental trials identified the trait as stress adaptive. While certain “extreme” staygreen phenotypes may be agronomically unsuitable, obtaining such knowledge helps identify environmental niches for which adoption of staygreen traits could provide maximum benefit.

## Conclusion

Improving our understanding of senescence requires adoption of a whole-plant phenotyping approach. When mapping staygreen traits underpinned by *NAM-1* mutations, we found TT70 and MeanPed to be the most stable and discriminative metrics. Thumbnail impressions can effectively detect variation in grain maturity associated with senescence traits, providing a rapid means of assessment. In combination, we hope that these insights can help qualify senescence traits, and aid identification and selection of senescence variation for both breeders and researchers alike.

## Data Availability Statement

Publicly available datasets were analyzed in this study. Phenotypic data reported in this publication relates to mapping of NAM-1 mutations corresponding to publication Chapman et al. (2020).

## Author Contributions

EAC, JL, and SG conceived the study. EAC designed and conducted the experiments with supervision from SG and JL. SO developed and maintained RIL populations. Contributions made by JL aided trait assessment and quantification performed by EAC. EAC analyzed the data and wrote the article in correspondence with SG and JL. All authors contributed to the article and approved the submitted version.

## Conflict of Interest

JL was employed by company KWS UK Limited. The remaining authors declare that the research was conducted in the absence of any commercial or financial relationships that could be construed as a potential conflict of interest.

## Supplementary Material

Supplementary Figure 1 Mean daily rainfall and temperature data for field seasons 2016–2018.

Supplementary Table 1 Year–pairwise senescence metric Spearman’s rank correlations for Staygreen A and Staygreen B RILs.
